# Ethical barriers to artificial intelligence in the national health service, United Kingdom of Great Britain and Northern Ireland

**DOI:** 10.2471/BLT.19.237230

**Published:** 2020-02-28

**Authors:** Claire Louise Thompson, Heather May Morgan

**Affiliations:** a School of Medicine, Medical Sciences and Nutrition, University of Aberdeen, Polwarth Building, Foresterhill, Aberdeen, AB25 2ZD, Scotland.

Artificial intelligence, the ability of systems to replicate human behaviour in an intelligent manner, shows promise in the United Kingdom of Great Britain and Northern Ireland’s National Health Service (NHS), which provides free-at-the-point-of-service health care via a national insurance scheme (Fig. 1). Recent advancements in artificial intelligence have created sophisticated software programmes that could revolutionize the NHS. Breakthroughs in machine learning, more notably deep learning (Box 1), have led to algorithms capable of performing diagnostic skills equivalent to those of doctors, automating administrative tasks and assisting in complex treatment management.

**Fig. 1:**
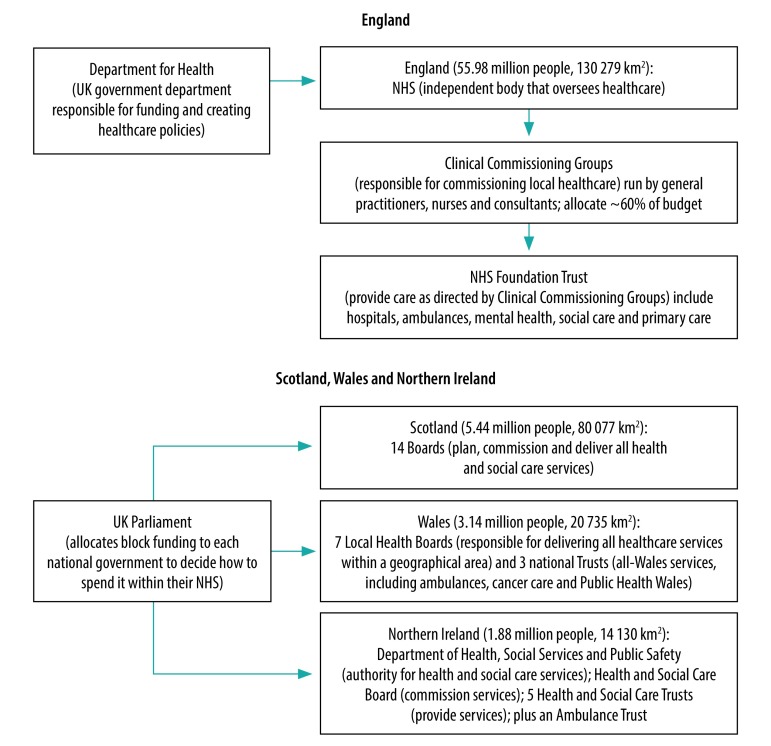
The structure of the National Health Service in the United Kingdom of Great Britain and Northern Ireland

NHS: National Health Service; UK: United Kingdom

Sources: National Health Service of England, Scotland, Wales and Northern Ireland websites: https://www.england.nhs.uk/; https://www.scot.nhs.uk/; https://www.wales.nhs.uk/; http://online.hscni.net/. Most recent national census data (2011) for each of the constituent of the United Kingdom: https://www.ons.gov.uk/.

Box 1 **Definition of machine learning and deep learning**

**Machine learning**

A subset of artificial intelligence that allows programmes to learn without being explicitly programmed. These programmes learn from data sets, identify patterns within them and use the information to make predictions about data they have not been previously exposed to.

**Deep learning**

A relatively new subset of machine learning, which uses algorithms that replicate the human brain (neural networks) in structure and function. In machine learning, the more data the algorithm can use, the greater its performance. In older styles of machine learning, algorithms eventually reach a plateau in performance, regardless of how much additional data is used to train the algorithm. Deep learning, however, can use greater quantities of data leading to higher levels of accuracy in algorithms that were not previously achievable, this is one of the main reasons deep learning has revolutionized the artificial intelligence field today. Despite the advantages of increased performance, one of the major drawbacks of deep learning is the lack of transparency in the algorithm’s operation (known as a ‘black box’ system). In deep learning, viewing the input and output of algorithms is possible; however, it is difficult, even for developers, to determine how the algorithm produced its output. This difficulty proves significantly problematic in disciplines such as health care, where justification for decisions reached is crucial to ensure maximum safety for patients.

As a result of the vast linkable data that the NHS holds on all citizens throughout their lives, the service could have a leading role in taking forward artificial intelligence development for health care;^1^ however, its use remains limited, with little overarching policy guiding its development and application. In 2018, the government of the United Kingdom published a code of conduct outlining expectations for artificial intelligence development in the NHS, covering aspects such as the appropriate handling of data, the need for algorithmic transparency and accountability.^2^ The code states that, in combination with the *conformité européenne* (CE) mark certification, health research and relevant regulatory approvals, it should provide an overall policy and structure for the creation of safe and effective artificial intelligence. The code, however, is only in its initial consultation stage. This paper discusses the issues highlighted within the code of conduct and the ethical challenges associated with addressing them to successfully integrate artificial intelligence within the NHS.

## Patient data in training algorithms

Daily, the NHS collects and records vast quantities of data, providing a valuable opportunity for methods requiring training through large data sets, such as machine learning.^3^ Patient data, however, could be potentially misused, and must be subject to relevant ethical and legal considerations by both developers and within the NHS.

A recent study found that 51% (1020/2000) of people surveyed in the United Kingdom were concerned about their data privacy as the use of artificial intelligence increases; this finding was particularly relevant for those with less knowledge about artificial intelligence capabilities.^4^ As part of the government’s code of conduct, complying with appropriate legal and ethical frameworks is required to safeguard patients. However, the growth of machine learning and its need for real-life data could present an ethical dilemma where patient data are being used as an exploitable resource for purposes other than those for which the data were originally collected. This situation has already occurred. The Royal Free NHS Trust failed to obtain appropriate consent for the use of data from 1.6 million patients in the development of one of its artificial intelligence applications.^5^ Repeated episodes such as this one could decrease public trust in the NHS handling of patient data and might make patients refuse to share their information. Ensuring that patients have explicitly consented to the use of their personal data in artificial intelligence development is therefore crucial. The introduction of a national data opt-out programme for patient information in 2018^6^ has made it easier for patients to control the use of their data. Additionally, guaranteeing that patient information continues to be viewed as a valued asset subject to appropriate ethical and legal considerations, as opposed to a free-for-all for use in training algorithms, is necessary.

## Transparency and accountability

Nearly all artificial intelligence software will fail to perform its intended purpose at some point during its lifetime.^7^ Although in some sectors, artificial intelligence failures can be trivial, failures in the health-care sector can have catastrophic consequences. Therefore, the ability to hold the responsible party accountable is vital. However, the assignment of accountability in artificial intelligence, and specifically machine learning, can be challenging, primarily due to the lack of transparency. Some studies suggest that humans may no longer be in control of what decision is taken and may not even know or understand why a wrong decision has been taken, because transparency is lost.^7^

Currently, artificial intelligence within the NHS is a tool to support staff rather than a decision-maker, meaning medical professionals are held accountable for decisions regardless of whether their decisions were influenced by artificial intelligence technology or not. The fact that NHS professionals can be held accountable for decisions influenced by potentially inaccurate artificial intelligence, which cannot be proven in some situations due to lack of transparency, may deter them from embracing the technology. Although the code of conduct highlights the importance of transparency, at present, ensuring this transparency may not be entirely possible and, if artificial intelligence is to take on a greater role in supporting clinicians in decision-making, the issue must be addressed.

The development of transparent machine learning techniques would benefit the accountability dilemma in artificial intelligence and address the previously mentioned regulatory issues, and therefore is an important area for study. Tackling transparency in artificial intelligence, specifically machine learning, can be challenging; however, research suggests it could be possible. Scientists have recently identified methods for developing transparent deep learning neural networks.^8^ Furthermore, developers tackled transparency issues, while improving artificial intelligence software for analysing ophthalmologic images, by displaying selected information regarding how the software arrived at its recommendation.^9^ Despite these promising examples, technological solutions to making machine learning transparent are still in their infancy and will require developers with expertise in this fast-moving field.

## Public trust

One of the key messages highlighted throughout the code of conduct is that gaining public trust is a high priority and crucial to artificial intelligence’s successful deployment within the NHS. Current reports, however, suggest that the NHS is in a less than desirable position, with a recent poll finding that only 20% (400/2000) of respondents support the use of artificial intelligence in health care,^4^ demonstrating the scale of the problem. Although there are numerous artificial intelligence success stories from recent years, these have arguably been outshined by catastrophic failures, which have inevitably dented the public’s already limited trust in the use of artificial intelligence. High-profile incidents, in combination with unfamiliarity and a lack of understanding present a significant problem.

Public trust in the use of artificial intelligence is essential for its growth, and relevant parties, including the government, must adopt measures to gain this trust. Efforts need to be made not only to ensure that patients are sufficiently empowered with education on current technology, but also reassured that the technology is safe and developed according to relevant technical and ethical standards. 

## Outlook

Ultimately, the use of artificial intelligence, especially machine learning, within the NHS has the potential to significantly improve patient care if measures are put in place to address current barriers. Due to the significant volume of patient data required in artificial intelligence development, developers and providers must adhere to data regulation and control to avoid inappropriate data exploitation. Data use must be subject to relevant ethical and legal considerations, which will require research attention from social scientists, philosophers and bioethicists working in applied health sciences and lawyers collaborating with health-care innovation teams, and providers to secure appropriate permissions and safeguards. A more joined-up, cross-disciplinary working model between academics and lawyers alongside NHS partners (technical and clinical) is needed at the earliest stage of every project to inform implementation. Addressing data use could diminish transparency and accountability concerns by ensuring the logic behind algorithms is available and clear. Training and capacity building in the technical aspects is needed to create expertise in how to investigate and make sense of system failure. Such focus within the discipline is improving and those working on this area of computing sciences will need to develop robust and transparent practices, and identify means of explaining technical processes to non-experts. An updated code of conduct is needed to guide practices and/or a benchmark gold standard codebreaker process. An example of such work is the Realizing Accountable Intelligent Systems project, an academic collaboration to develop auditing systems for artificial intelligence. Finally, with low levels of public trust in artificial intelligence, educating the public, ensuring that artificial intelligence policy is accessible and providing reassurance that software has been developed according to regulatory and safety standards, are needed. The growth of artificial intelligence within the NHS requires political attention to address current policy limitations and gaps within NHS trusts, and may also benefit from nation-wide unity on how to approach artificial intelligence use across the NHS. Academic research, including embedded stakeholder engagement, will be required to inform the development of such policies, and advocacy will be needed to promote these policies, using a range of science communication approaches for multiple public audiences.^10^ Although it may be some years before the NHS is fully able to use the potential of artificial intelligence, it is time to prepare for its growth.
